# Angina Bullosa Hemorrhagica of the Oral Mucosa: A Case Report

**DOI:** 10.7759/cureus.24648

**Published:** 2022-05-01

**Authors:** Rahul Navab, Visweswara R Yeragudi Jangamareddy

**Affiliations:** 1 Internal Medicine, People’s Education Society Institute of Medical Sciences and Research, Kuppam, IND

**Keywords:** oral mucous membrane, airway obstruction, oropharynx, blood-filled blisters, angina bullosa hemorrhagica (abh)

## Abstract

Angina bullosa hemorrhagica (ABH) is a condition of the oral mucous membrane, characterized by the sudden appearance of blood-filled blister(s) within the oral cavity. In the majority of cases, these blisters occur on the oropharynx or palate. The blisters usually rupture in a day or two and heal spontaneously without any further scarring or discomfort. In rare cases, if a large lesion located in the throat does not rupture spontaneously, it may lead to airway obstruction. We present the case of a 64-year-old-female who presented with a recurrent manifestation of well-defined oral blood-filled blisters which ruptured and healed spontaneously. There were no identifiable risk factors. Angina bullosa hemorrhagica was diagnosed clinically. The main objective of this case report is to bring awareness and avoid unnecessary investigations and misdiagnosis.

## Introduction

Angina bullosa hemorrhagica (ABH) is a rare disorder characterized by recurrent occurrences of blood-filled vesicles and bullae in the oral cavity, which are not caused by blood dyscrasia, immunobullous disorders, systemic diseases, or other causes [1]. The etiology is usually multifactorial or unknown. Blisters heal spontaneously without any scar formation. As early as 1933, Balina of Argentina reported the same lesions as traumatic oral haemophlyctenosis, and he also suggested that the lesions were due to trauma, especially among patients with senile capillary changes [2].

## Case presentation

**Figure 1:**
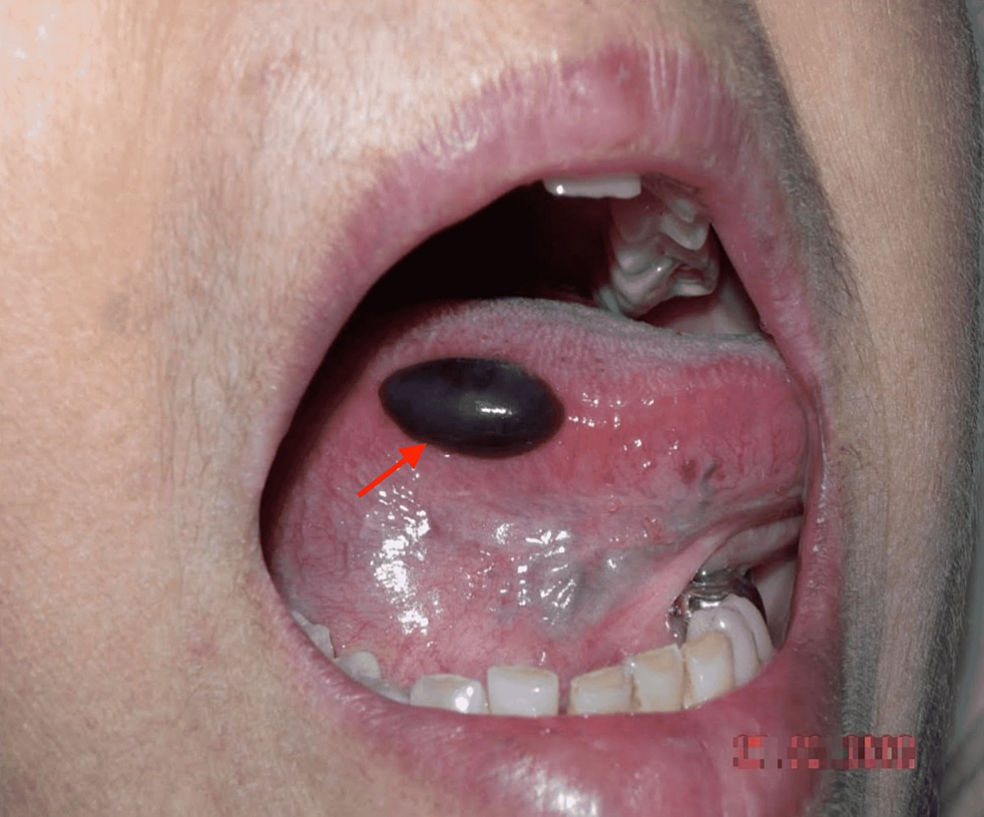
Well-defined, blood-filled blister on the right lateral aspect of the tongue as shown by red arrow

A 64-year-old-female presented to the hospital with a history of sudden onset of blister over the tongue. A well-defined hemorrhagic bulla of about 2 cm in diameter was found on the right lateral aspect of the tongue (Figure 1). She recalled that she has had similar recurrent lesions in the past, sometimes over the palate and throat as well, and recurring two to three times a year since childhood. All such lesions healed after rupture, without residual effects. She had no bleeding disorder or hemangioma in the past. The patient had no associated comorbid illness, and she also denied a history of any relevant risk factors like trauma, ingestion of spicy food, or incriminating drugs. Her vital signs were in the normal range. Systemic examination was normal. Routine hematological investigations and coagulation workup were within the normal range. A diagnosis of ABH was made based on clinical findings. The sudden onset of similar solitary and occasionally multiple blood blisters on the tongue, soft palate, and at the oral mucosa of the cheek during previous occurrences at different times was strongly suggestive of ABH. The blister resolved without any treatment.

## Discussion

ABH was first described by Badham in 1967 as blood-filled blisters in the oral, pharyngeal, and esophageal mucosa [3]. ABH is a rare benign condition characterized by the sudden appearance of well-defined, blood-filled blisters in the oral cavity, which rapidly expand and rupture spontaneously between 24 to 48 hours after their onset [4]. The etiopathogenesis of the disease is currently unknown and therefore considered multifactorial [5]. Elderly and middle-aged people are affected commonly, and lesions may be multiple or solitary [6]. The most commonly affected site is the soft palate [1]. In our case, the lesion was a non-tender, well-defined, blood-filled blister about 2 cm in diameter over the right lateral aspect of the tongue. Clinically, patients may rarely experience hoarseness of voice or bloody sialorrhea when this lesion occurs over the soft palate or throat [7]. ABH can occur suddenly by oral trauma and usually resolves spontaneously within three to 10 days after its onset. Risk factors include trauma to the oral mucosa, eating hot spicy food, inhaling steroids, and diabetes mellitus.

In our case, the patient reported no risk factors. She denied a history of any vascular lesion or hemangioma in the past. Laboratory workup of blood counts, platelets, and coagulation panel was in the normal range. Biopsy was not necessary in our case as a confident diagnosis of ABH was clinically obvious after ruling out other hematological and mucosal diseases based on routine investigations work-up. Disorders of collagen and elastic fibers in the oral mucosa can reduce the anchoring of the blood vessels, causing hemorrhagic lesions after trauma leading to these pathologic conditions in patients [8]. The presentation of this benign disorder must be distinguished from other more serious disorders, like Kaposi sarcoma or epidermolysis bullosa acquisita, with similar presentations [9]. Other vascular lesions or hemangiomas were not considered because our patient had sudden onset of the blood-filled blister. As seen in our case, lesions may reoccur clinically, and the diagnosis of ABH is largely clinical [10]. 

A few differential diagnoses like bullous pemphigoid, pemphigoid, bullous lichen planus, dermatitis herpetiformis, epidermolysis bullosa, oral amyloidosis, and thrombocytopenia need to be considered [1]. Angina bullosa hemorrhagica should be distinguished from other subepithelial bullous lesions that involve the oral cavity [11]. Based on the detailed clinical history, clinical examination, blood investigations, and histopathological study to exclude other conditions, one can diagnose ABH. 

The following nine diagnostic criteria for ABH were introduced by Ordioni et al. (Table 1) [12].

**Table 1:** Nine diagnostic criteria presented by Ordioni et al. [12] ABH - angina bullosa hemorrhagica

ABH can be diagnosed based on the following nine criteria
A) Hemorrhagic blister or erosion that is clinically visible in the oral mucosa that has a history of bleeding
B) Localization exclusively oral or oropharyngeal
C) Palatal location
D) A triggering event or promoting factor (food intake)
E) Recurrent lesions
F) Favorable evolution without a scar within a few days
G) Painless lesion, or tingling or burning sensation
H) Platelet count and coagulation parameters should be within normal ranges
I) Results of direct immunofluorescence are negative

In this case, six out of nine (including main criteria A and B) of the above-mentioned diagnostic criteria were met by the patient. In general, no active treatment is needed as it can heal spontaneously. If the ABH has affected the soft palate and the blister has ruptured, antibiotic prophylaxis and antiseptic rinses are recommended, such as chlorhexidine digluconate at a concentration of 0.25% to 0.12%, to help prevent secondary infections [13]. An intact large palatal or pharyngeal blister causing a feeling of choking should be surgically treated [2]. ABH carries a good prognosis.

## Conclusions

Since this is a rare disorder, many are unaware of this condition, which results in unnecessary investigations and misdiagnosis. ABH can be diagnosed confidently by its classic clinical presentation after ruling out other causes of blood-filled bullous lesions of the oral cavity. This case report creates awareness about ABH among the medical fraternity. Simple reassurance and counseling of the patient is sufficient to manage this condition.
